# A novel biomarker associated with EBV infection improves response prediction of immunotherapy in gastric cancer

**DOI:** 10.1186/s12967-024-04859-8

**Published:** 2024-01-22

**Authors:** Xiaoqin Li, Fen Xiong, Zhangmin Hu, Qing Tao, Yufei Yang, Xuehan Qiao, Chen Peng, Yuchun Jiang, Miao Han, Kebin Dong, Yi Hua, Wei Zhang, Min Xu, Weiguo Long, Yichuan Xiao, Deqiang Wang

**Affiliations:** 1 Department of Oncology, Digestive Disease Institute&Cancer Institute of Jiangsu University, Affiliated Hospital of Jiangsu University, Zhenjiang, 212001 China; 2 Department of Gastroenterology, Digestive Disease Institute of Jiangsu University, Affiliated Hospital of Jiangsu University, Zhenjiang, 212001 China; 3 Department of Pathology, Affiliated Hospital of Jiangsu University, Zhenjiang, 212001 China; 4 CAS Key Laboratory of Tissue Microenvironment and Tumor, Shanghai Institute of Nutrition and Health, University of Chinese Academy of Sciences, Chinese Academy of Sciences, Shanghai, 200031 China

**Keywords:** Epstein-Barr virus, Immunotherapy, Gastric cancer, Biomarker

## Abstract

**Background:**

Novel biomarkers are required in gastric cancer (GC) treated by immunotherapy. Epstein-Barr virus (EBV) infection induces an immune-active tumor microenvironment, while its association with immunotherapy response is still controversial. Genes underlying EBV infection may determine the response heterogeneity of EBV + GC. Thus, we screened hub genes associated with EBV infection to predict the response to immunotherapy in GC.

**Methods:**

Prognostic hub genes associated with EBV infection were screened using multi-omic data of GC. EBV + GC cells were established and confirmed by EBV-encoded small RNA in situ hybridization (EBER-ISH). Immunohistochemistry (IHC) staining of the hub genes was conducted in GC samples with EBER-ISH assay. Infiltrating immune cells were stained using immunofluorescence.

**Results:**

CHAF1A was identified as a hub gene in EBV + GC, and its expression was an independent predictor of overall survival (OS). EBV infection up-regulated CHAF1A expression which also predicted EBV infection well. CHAF1A expression also predicted microsatellite instability (MSI) and a high tumor mutation burden (TMB). The combined score (CS) of CHAF1A expression with MSI or TMB further improved prognostic stratification. CHAF1A IHC score positively correlated with the infiltration of NK cells and macrophages M1. CHAF1A expression alone could predict the immunotherapy response, but its CS with EBV infection, MSI, TMB, or PD-L1 expression showed better effects and improved response stratification based on current biomarkers.

**Conclusions:**

CHAF1A could be a novel biomarker for immunotherapy of GC, with the potential to improve the efficacy of existing biomarkers.

**Supplementary Information:**

The online version contains supplementary material available at 10.1186/s12967-024-04859-8.

## Background

Gastric cancer (GC), which is known for its high heterogeneity and treatment complexity, ranks fifth in terms of morbidity and third in terms of mortality worldwide among all malignant tumors [1]. In recent years, immunotherapy using immune checkpoint inhibitors (ICIs), mainly against programmed cell death receptor-1 (PD-1) or its ligand, PD-L1, has been one of the biggest advances in the treatment of GC. In particular, in the first-line treatment of metastatic HER2-negative GC, pivotal phase III trials, such as CheckMate 649, KEYNOTE-859, and ORIENT-16, reported that the combination of ICIs with chemotherapy significantly improved overall survival (OS) and progression-free survival (PFS) compared with chemotherapy alone [2]. However, in both the CheckMate 649 [3] and ORIENT-16 trials [4], the survival benefit of immunotherapy seems to come mainly from patients with PD-L1 combined positive score (CPS) ≥ 5, whose proportions (60 and 61%, respectively) were substantially higher than other reports, and such benefit is still controversial in patients with CPS < 5 [5]. In addition, immunotherapy in second/third-line therapies of GC still has a very limited efficacy (approximately 10% for monotherapy response), regardless of PD-L1 expression [6, 7]. Novel biomarkers can help in further identifying patients who may benefit from ICI treatment, particularly in the second-/third-line setting.

According to molecular characterization by The Cancer Genome Atlas (TCGA), GC can be classified into four molecular subtypes: chromosomal instability (CIN), Epstein-Barr virus-positive (EBV +), genomically stable (GS), and microsatellite instability (MSI) GC [8]. EBV + GC accounts for 2–20% of all GC cases and is characterized by an EBV infection, which is usually detected using EBV-encoded small RNA in situ hybridization (EBER-ISH) [9]. Clinically, EBV testing is often performed based on an undifferentiated phenotype observed by pathologists, described as lymphoepithelioma-like or medullary, and characterized by a dense infiltrate of lymphocytes [10]. Compared to EBV- GC, EBV + GC has an immune-active tumor microenvironment [11]. Recently, a phase II trial reported that EBV + GC dramatically responded to second-line immunotherapy with pembrolizumab, with an overall response rate (ORR) of 100% (six patients) [12]. In contrast, another phase II trial which also enrolled six patients with EBV + GC observed no response to salvage treatment with camrelizumab [13]. These results indicate that EBV + GC remains highly heterogeneous. In a retrospective study, PD-L1 expression further stratified the outcomes of patients with EBV + GC treated with ICIs [14]. Such heterogeneity is also reflected by the genomic alterations underlying EBV infection [15].

In this study, we screened hub genes associated with EBV + GC to identify novel biomarkers of EBV infection and immunotherapy efficacy. We found that CHAF1A, a histone chaperone, was upregulated upon EBV infection. The combination of CHAF1A and current immunotherapeutic biomarkers has the potential to improve clinical practice.

## Methods

### GC patients with EBV infection data

GC patients diagnosed between January 2020 and August 2023 at the Affiliated Hospital of Jiangsu University (AHJU) were screened for information regarding EBV infection through the following eligibility criteria: gastrectomy, EBER-ISH detection, sufficient tissue for immunohistochemistry (IHC), pathological diagnosis of gastric adenocarcinoma, and no prior history of anticancer therapy (including neoadjuvant therapy). The American Joint Committee on Cancer criteria were used for the clinical and clinicopathological classification and staging. Approval was obtained from the ethics committee of AHJU prior to the study.

Three other GC cohorts with EBV infection data were also used, including those from TCGA [8], Asian Cancer Research Group (ACRG) [16], and NCT#02589496 phase II trial [12]. TCGA and ACRG cohorts were used to screen EBV-associated hub genes and validate their prognostic roles. The AHJU and NCT#02589496 cohorts were used to confirm the association between the hub genes and EBV infection.

### Immunotherapy patients

Two cohorts were used to investigate the role of the target gene in the prediction of immunotherapy outcomes. The NCT#02589496 GC cohort enrolled patients to receive second/third-line treatment with pembrolizumab [12]. The IMvigor210 cohort [17] included patients with metastatic urothelial cancer (mUC) to receive second-line atezolizumab therapy.

### Multi-omic data

Transcriptome data from 34 patients in an additional AHJU GC cohort were used to explore the signaling network associated with the object gene [18, 19]. Transcriptome data were stored in the European Genome-Phenome Archive (https://ega-archive.org/), with the identification number EGAD00001004164. Data from other cohorts, including mRNA expression, EBV infection status, tumor mutation burden (TMB), tumor neoantigen burden (TNB), MSI, microsatellite stability (MSS), PD-L1 CPS, and clinical data, have been previously published and were acquired and preprocessed as described elsewhere [19, 20].

### Screening of hub genes associated with EBV + GC

The differentially expressed genes (DEGs) were determined between EBV + GC and EBV- GC using the *limma* R package in TCGA cohort, with log_2_(fold change) > 0.5 and *p* < 0.0001. The prognostic role of the DEGs was evaluated using univariate Cox proportional hazards models, and hazard ratios (HRs) with 95% confidence intervals (CIs) were calculated. DEGs with a significant prognostic impact (*p* < 0.001) that were consistent between ACRG and TCGA cohorts were selected. Next, we evaluated the degree of association between DEGs based on semantic similarities in their molecular functions in Gene Ontology (GO) and ranked DEGs based on the average functional similarities between the gene and its interaction partners [21]. The higher the average functional similarity, the more genes associated with it and the more significant the tested gene.

### Cell lines

The human GC cell line HGC-27 and the EBV virus-transformed monkey lymphocyte line B95-8 were purchased from the Type Culture Collection of the Chinese Academy of Science (Shanghai, China).

### Generation of EBV + GC cells

B95-8 cells were centrifuged, precipitated, and resuspended in a fresh culture medium. When HGC-27 cells grew to 50% of the culture vessel, B95-8 cells were added and gradually layered on the adherent HGC-27 cell layer from the suspension so that the two cell types began to contact and co-culture. After incubation for 24 h, anti-IgM antibodies and fresh rabbit serum were added to remove B95-8 cells via the immune toxicity response activated by the complement system.

### EBER-ISH

ISH was performed using an EBER kit (Zhongshan Jinqiao Biotechnology Co., Ltd.) with an EBER probe according to the manufacturer’s instructions. Briefly, cells were inoculated into a chamber culture slide, fixed with formalin, dehydrated with ethanol after 24 h of culture, and incubated overnight with an EBER probe labeled with digoxin. Diaminobenzidine was used for visualization.

### Western blot (WB) and RT-PCR

WB was performed using an anti-CHAF1A (ab126625, Abcam, UK) antibody according to standard protocols. Briefly, after extraction and quantification, total proteins were separated by SDS-PAGE and subsequently transferred onto PVDF membranes (Millipore, Bedford, MA, USA). Then, the membranes were blocked with 5% nonfat dry milk and incubated with ab126625 overnight at 4 °C. Finally, immunoblots were probed with ECL detection reagent (Millipore).

RT-PCR analysis of cDNA was performed using GoTaq qPCR Master Mix and an ABI7300 instrument (Applied Biosystems, USA) according to the manufacturer’s instructions. Briefly, TRIzol (Invitrogen, USA) was used to prepare total RNA, and the Access Reverse Transcriptase-PCR System (Promega, USA) was used to synthesize cDNA.

### IHC and multiple-immunofluorescence (mIF) staining

IHC was performed using an anti-CHAF1A antibody (ab126625), with a 2-step protocol. Specialized pathologists calculated the number of positively stained cells and the staining intensity to create grade categories under a microscope. A previously reported semi-quantitative method was used to assess IHC scores [22]. mIF staining was conducted using the PANO 7-plex IHC kit (Panovue, Beijing, China), section images were reconstructed using the Mantra System (PerkinElmer, Waltham, MA, USA), and quantification of cells in the images was performed using the inForm image software (PerkinElmer). Anti-CD8 (CST70306; Cell Signaling Technology, USA), anti-CD56 (CST3576), anti-CD68 (BX50031; Biolynx, China), anti-HLA-DR (ab92511), anti-panCK (CST4545), and anti-S100 (ab52642) antibodies were used for staining.

### Combined score (CS)

In the survival analysis, the optimal cutoff value to define high and low subgroups of TMB or CHAF1A expression with the most significant survival difference was determined using the *Survminer* R package. The TMB value and CHAF1A expression level were converted to either 1 (high) or 0 (low). EBV infection, MSI, and PD-L1 CPS with a cutoff value of 1 or 5 (CPS1 or CPS5) were converted to either 1 (yes/high) or 0 (no/low). CS was defined as the sum of CHAF1A expression levels with TMB, EBV, MSI, CPS1, and CPS5, ranging from 0 to 2. For response prediction, the receiver operating characteristic (ROC) curve and area under the ROC curve (AUC) were used to evaluate the predictive power of TMB and CHAF1A expression, which was subsequently dichotomized into 1 (high) or 0 (low) based on the optimal threshold of the maximum ROC curve values. Finally, a model for response prediction including all biomarkers was constructed using binary logistic regression with the entry method.

### Statistical analyses

According to the need for comparisons between groups, χ^2^ test, Fisher’s exact probability test, Student’s *t*-test, and Mann–Whitney *U* test were adopted. The predictive power of *CHAF1A* mRNA expression for EBV infection was evaluated by ROC and AUC based on the *pROC* R package. HRs with 95% CIs were calculated to analyze the independent prognostic value of *CHAF1A* mRNA expression using multivariate Cox proportional hazard models. The Kaplan–Meier method with the log-rank test was used for survival analysis. Statistical significance was set at *p* < 0.05. SPSS (version 19.0, Chicago, IL, USA) and R (version 3.6.1) were used for all analyses.

## Results

### Clinical characteristics

EBER-ISH for EBV was performed in 34 GC cases at the AHJU between January 2020 and August 2023 because of a lymphoepithelioma-like or medullary phenotype. Twenty-six patients were eligible and included in this study, including five EBV + and twenty-one EBV- GC patients (Additional file 1: Table S1). The number of eligible patients in the ACRG, TCGA, and NCT#02589496 cohorts was 275, 349, and 45, respectively. Compared with EBV- GC, EBV + GC had more men in AHJU (71.4 vs. 100%, *p* = 0.173), ACRG (65.8 vs. 88.9%, *p* = 0.043), and TCGA (64.3 vs. 85.2%, *p* = 0.028). The proportion of histologic grade III/IV in EBV + GC decreased in AHJU (85.7 vs. 40%, *p* = 0.029) but increased in ACRG (56 vs. 83.3%, *p* = 0.023) and TCGA (56.9 vs. 92.6%, *p* < 0.001), indicating population heterogeneity.

### CHAF1A is a hub gene in EBV infection of GC

**Fig. 1:**
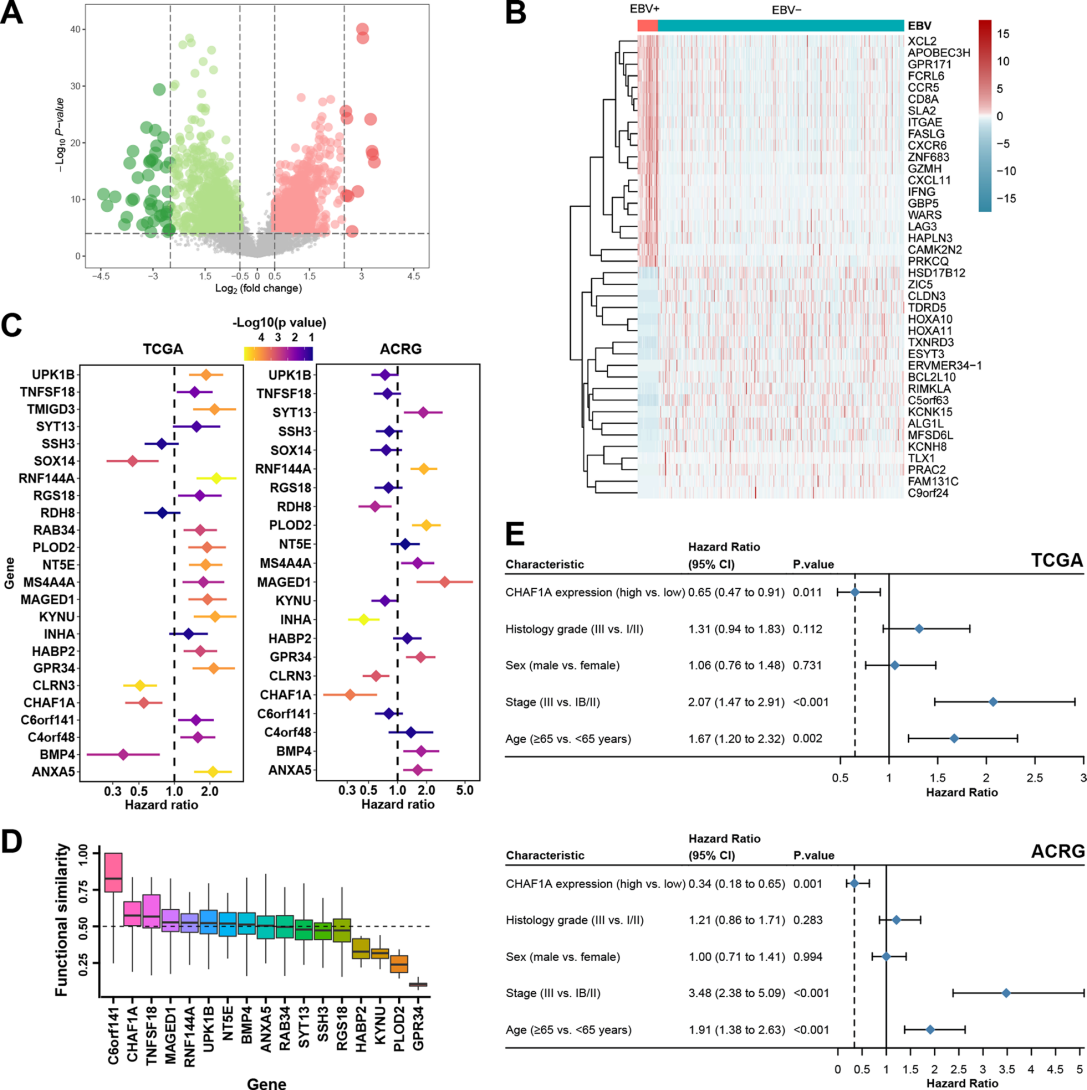
Selection of EBV-associated genes. **A**: Volcano plot for differentially expressed genes (DEGs) between EBV- and EBV + GC in the TCGA cohort. **B**: Heatmap of the TOP 40 DEGs. **C**: Prognostic DEGs in both the ACRG and TCGA GC cohorts. **D**: Average functional similarities between the gene and its interaction partners according to their semantic similarities of Gene Ontology terms for molecular function. **E**: CHAF1A mRNA expression is an independent predictor of overall survival in both the TCGA and ACRG cohorts. *GC* gastric cancer, *ACRG* Asian Cancer Research Group, *TCGA* The Cancer Genome Atlas

A total of 2,005 DEGs were determined between EBV + and EBV- GC from TCGA (Fig. 1A, B). Univariate Cox analysis revealed that the expression levels of the 24 genes were prognostic for OS in both the ACRG and TCGA cohorts (*p* < 0.001; Fig. 1C). Of these, *C6orf141* and *CHAF1A* ranked as the top two in terms of the number of interacting partner genes (Fig. 1D), whereas only *CHAF1A* had a consistent prognostic role between ACRG and TCGA. Furthermore, *CHAF1A* was identified as an independent OS predictor in both the ACRG (HR = 0.34, 95% CI 0.18–0.65, *p* = 0.001) and TCGA (HR = 0.65, 95% CI 0.47–0.91, *p* = 0.011) cohorts (Fig. 1E).

### EBV infection and CHAF1A mRNA expression

**Fig. 2:**
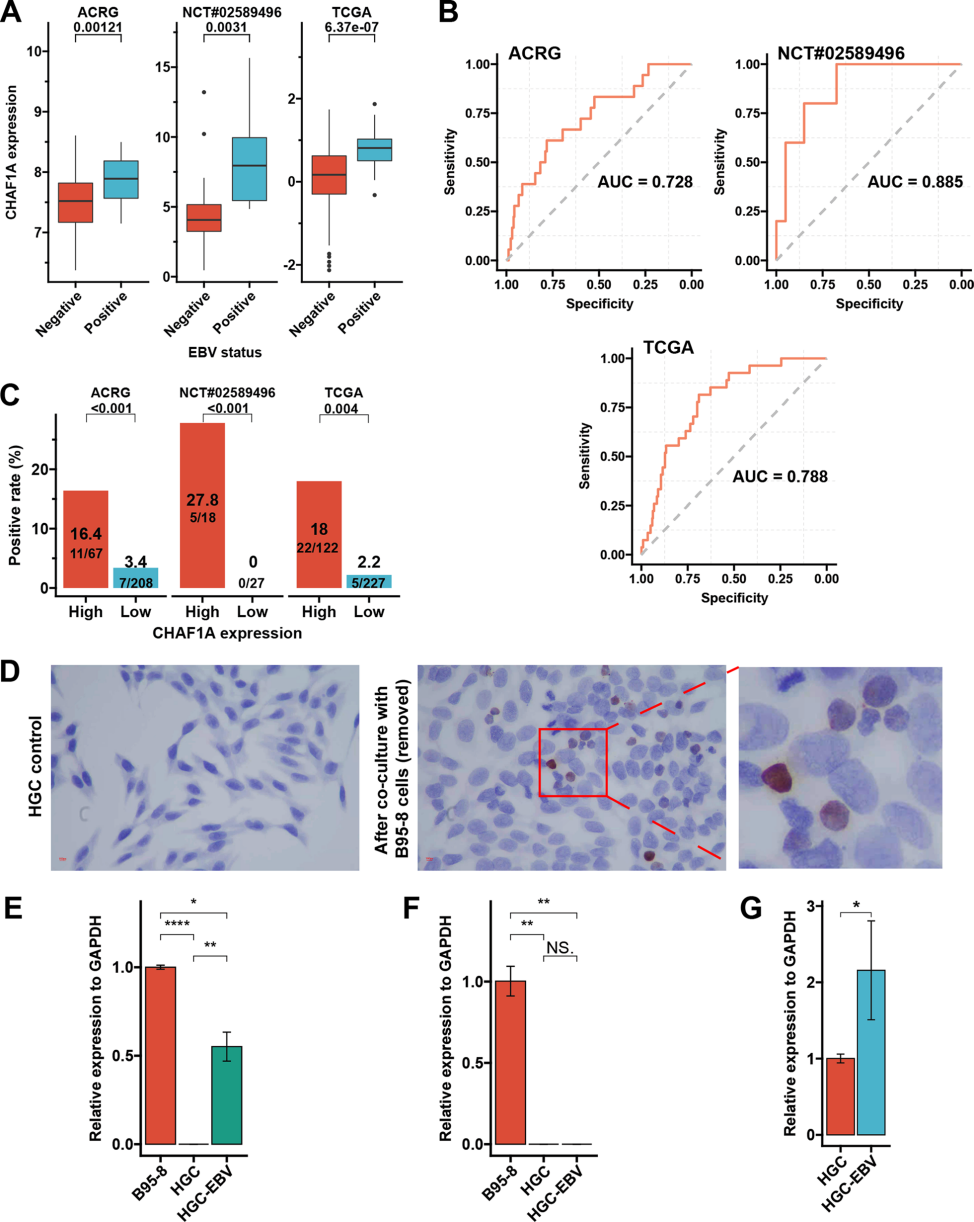
EBV infection and CHAF1A mRNA expression. **A**: CHAF1A mRNA expression according to EBV infection status. **B**: The ROC curve for EBV infection prediction by CHAF1A mRNA expression. **C**: Positive rate of EBV infection according to CHAF1A expression level. Based on the optimal threshold of CHAF1A expression for the maximum ROC curve values, the patients are dichotomized into high and low subgroups. **D**: EBV + HGC (HGC-EBV) cells (reddish brown) detected by EBER after co-culture of HGC with B95-8 cells (removed). **E**: HGC-EBV cells express BamHI-W mRNA which is exclusively expressed by EBV + cells. **F**: HGC-EBV cells do not express CAJA-DRB1 mRNA which is exclusively expressed by marmoset cells, indicating that the B95-8 cells have been removed. **G**: EBV infection improves CHAF1A mRNA expression. *AHJU* Affiliated Hospital of Jiangsu University, *ROC* receiver operating characteristic curve, *AUC* the areas under the ROC curves

In all the ACRG, NCT#02589496, and TCGA cohorts, *CHAF1A* mRNA expression was significantly higher in EBV + GC than that in EBV- GC (Fig. 2A). ROC analysis showed that the AUC for the prediction of *CHAF1A* mRNA expression in EBV infection were 0.728 (sensitivity:0.611; specificity:0.782), 0.885 (sensitivity:1.000; specificity:0.675), and 0.788 (sensitivity:0.815; specificity:0.689) in the three cohorts, respectively (Fig. 2B). Based on the optimal threshold of CHAF1A expression for the maximum ROC curve values in each cohort, patients were divided into high- and low-expression subsets. The incidence of EBV infection in the high- and low-expression subgroups was 16.4 and 3.4% (*p* < 0.001), 27.8 and 0% (*p* < 0.001), and 18 and 2.2% (*p* = 0.004), respectively (Fig. 2C). More importantly, EBV + HGC (HGC-EBV) cells were established (Fig. 2D–F), revealing that EBV infection significantly upregulated *CHAF1A* mRNA expression (Fig. 2G).

### EBV infection and CHAF1A protein expression

**Fig. 3:**
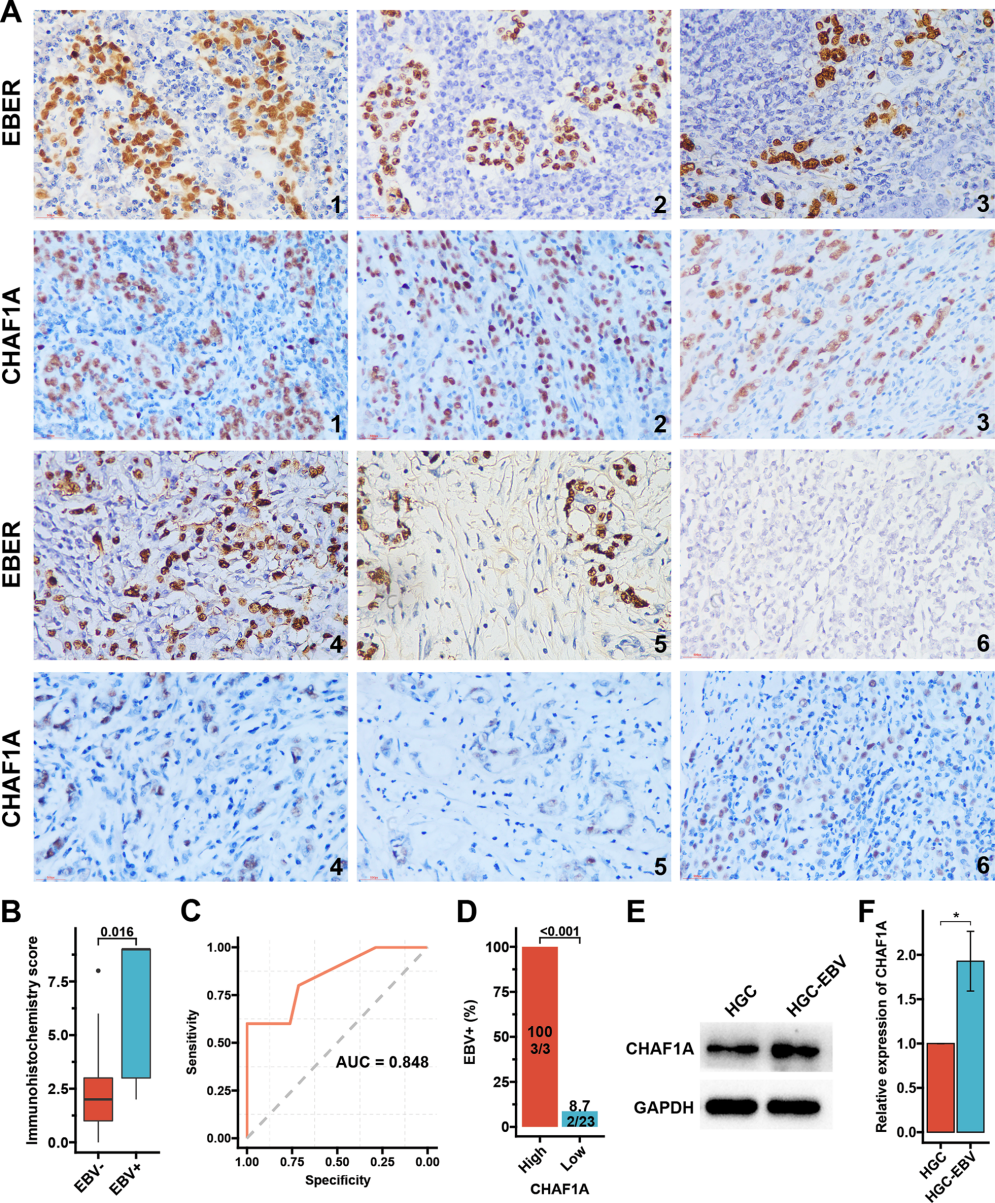
EBV infection and CHAF1A protein expression. **A**: EBER staining by in situ hybridizations and paired CHAF1A staining by IHC in five EBV-positive GC samples (No.1–5) and in one typical EBV-negative GC sample (No.6), respectively (typical micrograph at 200 × magnification). Because EBER was detected previously, the slice sections or the sampling tissues for CHAF1A detection are not the same as EBER. **B**: IHC score of CHAF1A between EBV + and EBV- samples. **C**: The ROC curve for EBV infection prediction by CHAF1A IHC score. **D**: Positive rate of EBV infection according to CHAF1A expression level. Based on the optimal threshold of CHAF1A IHC score for the maximum ROC curve values, the patients are dichotomized into high and low subgroups. **E**: EBV infection improves CHAF1A protein expression. **F**: Quantization results of grayscale values in (**E**). *IHC* immunohistochemistry, *ROC* receiver operating characteristic curve, *AUC* the areas under the ROC curves

IHC staining for CHAF1A was conducted on 26 GC samples from AHJU whose EBV infection status was available. Three of the five EBV + GC presented a strong CHAF1A staining (Fig. 3A), and the IHC score was significantly higher in EBV + GC than that in EBV- GC (*p* = 0.016; Fig. 3B). The AUC for predicting the CHAF1A IHC score for EBV infection was 0.848 (sensitivity:0.600; specificity:1.000; Fig. 3C). Based on the optimal threshold of the CHAF1A IHC score for the maximum ROC curve values, the patients were divided into high- and low-expression subsets. The incidence of EBV infection in the high- and low-expression subgroups was 100 and 8.7%, respectively (*p* < 0.001; Fig. 3D). More importantly, EBV + HGC cells had significantly upregulated CHAF1A protein expression, as detected by WB than EBV- HGC cells (Fig. 3E, F).

### CHAF1A-associated signaling involve many pathogen infections

**Fig. 4:**
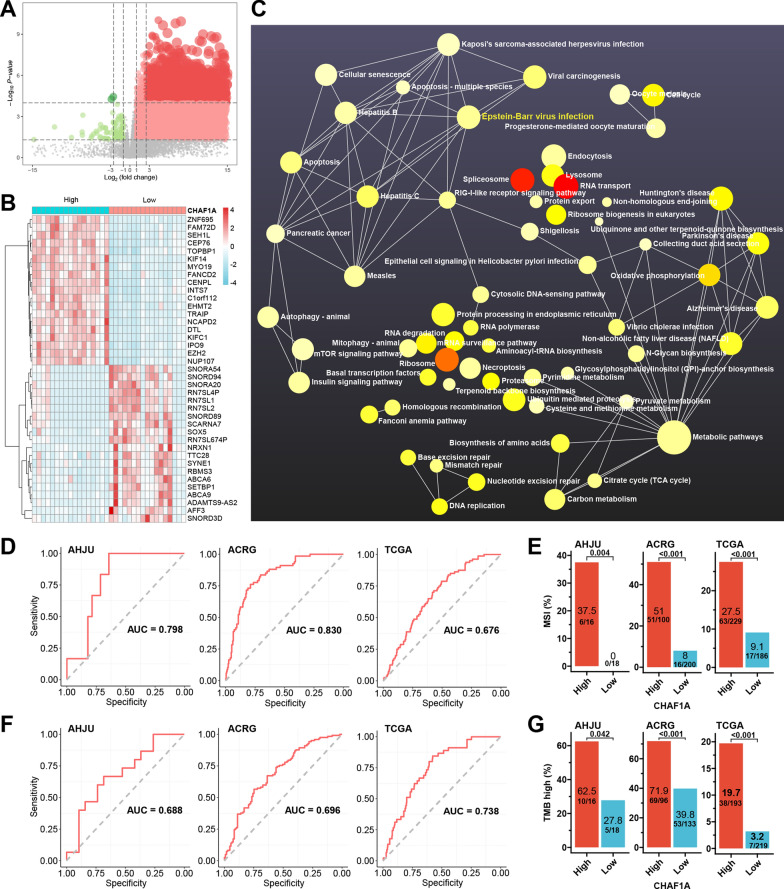
Signaling pathway associated with CHAF1A. **A**: Volcano plot for differentially expressed genes (DEGs) between high and low subgroup of CHAF1A expression in the AHJU cohort. **B**: Heatmap of the TOP 40 DEGs. **C**: Enrichment analysis for KEGG signaling pathway associated with CHAF1A. **D**: The ROC curves for MSI prediction by CHAF1A mRNA expression. **E**: MSI incidence according to CHAF1A expression level. Based on the optimal threshold of CHAF1A expression for the maximum ROC curve values, the patients are dichotomized into high and low subgroups. **F**: The ROC curves for TMB-high prediction by CHAF1A expression. The optimal cutoff value with the most significant survival difference was used to to define high and low TMB. **G**: TMB-high incidence according to CHAF1A expression level. *AHJU* Affiliated Hospital of Jiangsu University, *KEGG* Kyoto Encyclopedia of Genes and Genomes, *MSI* microsatellite instability, *TMB* tumor mutation burden, *ROC* receiver operating characteristic curve, *AUC* the areas under the ROC curves

In an additional AHJU GC cohort with transcriptome data from 34 patients [18, 19], DEGs were identified between the high and low subgroups (median value as the cutoff) of CHAF1A expression, based on the criteria of adjusted *p*-value < 0.05, and log_2_(fold change) > 1 (Fig. 4A, B). Then, NetworkAnalyst 3.0 (https://www.networkanalyst.ca/) and pathways in Kyoto Encyclopedia of Genes and Genomes (KEGG) were applied for gene set enrichment analysis (GSEA). The enriched pathways in the high-expression group involved many pathogen infection signaling pathways, including EBV infection, hepatitis B, hepatitis C, measles, shigellosis, *Vibrio cholerae* infection, epithelial cell signaling in *Helicobacter pylori* (HP) infection, Kaposi’s sarcoma-associated herpesvirus infection, and viral carcinogenesis (Fig. 4C). Other enriched pathways were associated with the regulation of gene expression, metabolic pathways, and DNA damage repair.

### CHAF1A is closely associated with MSI and TMB

GSEA indicated an association between CHAF1A and multiple DNA repair pathways, such as mismatch repair, base excision repair, nucleotide excision repair, and homologous recombination (Fig. 4C). Therefore, we investigated the association between CHAF1A, MSI, and high TMB, both of which are usually caused by abnormal DNA repair. In the AHJU, ACRG, and TCGA cohorts, the AUC for the prediction of CHAF1A mRNA expression for MSI were 0.798, 0.830, and 0.676, respectively (Fig. 4D). Based on the optimal threshold of CHAF1A expression for the maximum ROC curve values, patients were divided into high- and low-expression subsets. The MSI incidence between the high- and low-expression subgroups was 37.5 and 0% (*p* = 0.004), 51 and 8% (*p* < 0.001), and 27.5 and 9.1% (*p* < 0.001), respectively (Fig. 4E). Furthermore, the AUC for the prediction of CHAF1A expression for TMB-high (the optimal cutoff value with the most significant survival difference as the cutoff) were 0.688, 0.696, and 0.738 for these three cohorts, respectively (Fig. 4F). The incidence of TMB between the high- and low-expression subgroups of CHAF1A was 62.5 and 27.8% (*p* = 0.042), 71.9 and 39.8% (*p* < 0.001), and 19.7 and 3.2% (*p* < 0.001), respectively (Fig. 4G).

### Combination of CHAF1A expression with MSI or TMB improves prognosis stratification

**Fig. 5:**
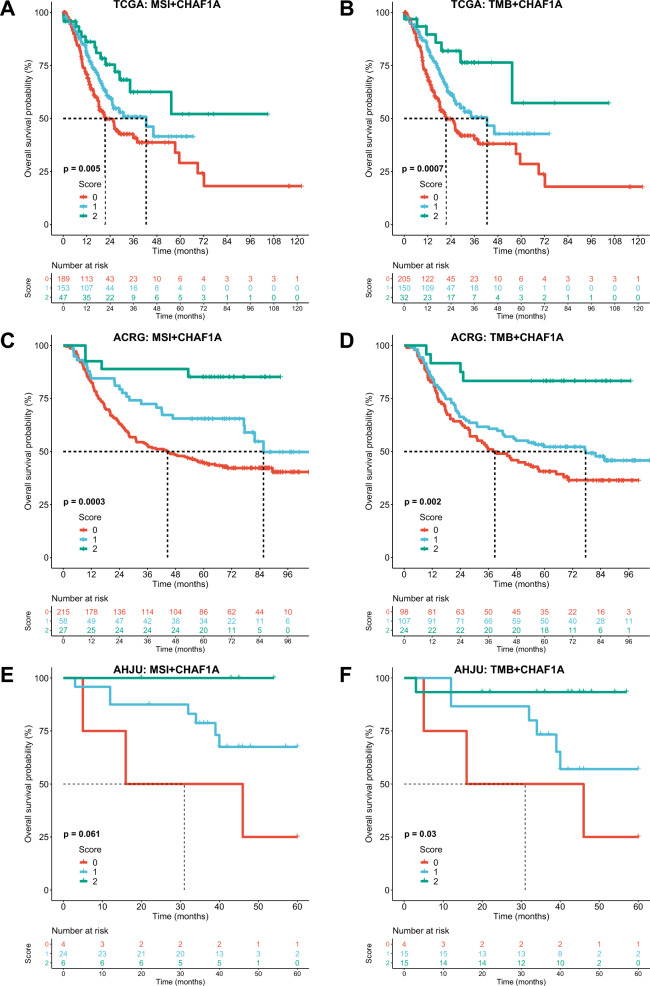
Overall survival stratified by the combined scoring of CHAF1A with classic biomarkers in gastric cancer. **A** and **B**: the combined scoring of CHAF1A with MSI (**A**) and TMB (**B**) in TCGA. **C** and **D**: the combined scoring of CHAF1A with MSI (**C**) and TMB (**D**) in ACRG. **E** and **F**: the combined scoring of CHAF1A with MSI (**E**) and TMB (**F**) in AHJU. *AHJU* Affiliated Hospital of Jiangsu University, *ACRG* Asian Cancer Research Group, *TCGA* The Cancer Genome Atlas, *MSI* microsatellite instability, *TMB* tumor mutation burden

According to the CS of CHAF1A expression with MSI or TMB, patients were stratified into three subgroups with scores of 0, 1, and 2. For the combination of CHAF1A with MSI, the median OS of patients with a score of 0, 1, and 2 were 21.4 months, 42.5 months, and not reached (NR) in TCGA (*p* = 0.005); 44.6 months, 85.6 months, and NR in ACRG (p = 0.0003); and 31.5 months, NR (seven events in 24 patients); and NR (no events in six patients) in AHJU (*p* = 0.061), respectively. For the combination of CHAF1A with TMB, the median OS of patients with a score of 0, 1, and 2 were 21.4 months, 42.5 months, and NR in TCGA (p = 0.0007); 37.9 months, 77.5 months, and NR in ACRG (p = 0.002); and 31.5 months, NR (6 events in 15 patients), and NR (1 events in 15 patients) in AHJU (p = 0.03), respectively (Fig. 5).

### CHAF1A correlates with immune cell infiltration

**Fig. 6:**
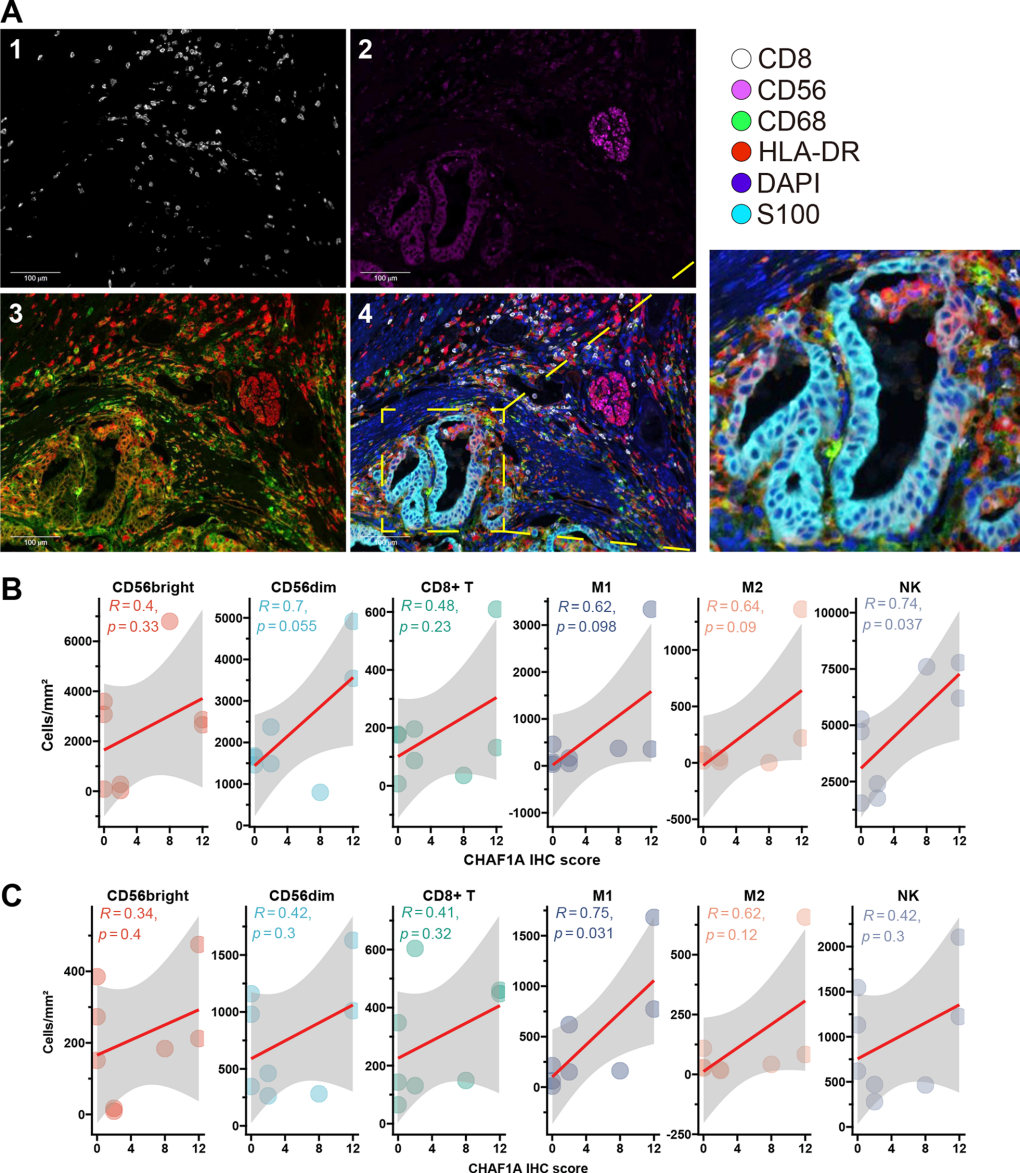
CHAF1A and immune cell infiltration. **A**: Typically microscopic image of multiple immunofluorescence staining for surface biomarkers of immune cells. 1: CD8; 2: CD56; 3: CD68 (green) and HLA-DR (red); 4: the reconstructed image for all biomarkers. **B** and **C**: Correlations between CHAF1A IHC score and the densities of immune cells in the tumor parenchyma (**B**) and stroma (**C**), respectively. IHC: immunohistochemistry

Since MSI and high TMB indicate a favorable immune microenvironment [23], we studied the association between CHAF1A expression and immune cell infiltration in GC. mIF staining of immune cells (Fig. 6A) and IHC staining of the CHAF1A protein (Fig. 3A) were simultaneously performed in eight GC samples. Cells in the tumor parenchyma and stroma were quantified separately. The CHAF1A IHC scores positively correlated with the density of NK cells (Pearson R = 0.74, *p* = 0.037; Fig. 6B) in the tumor parenchyma and macrophages M1 in the stroma (R = 0.75, *p* = 0.031; Fig. 6C). A positive correlation was also observed between the CHAF1A IHC score and the densities of other cells, including CD8 + T cells, macrophages M2, the CD56_bright_ NK subset, and the CD56_dim_ NK subset, although significance was limited by the small sample size.

### Combination of CHAF1A expression with classic biomarkers improves response prediction of immunotherapy

**Fig. 7:**
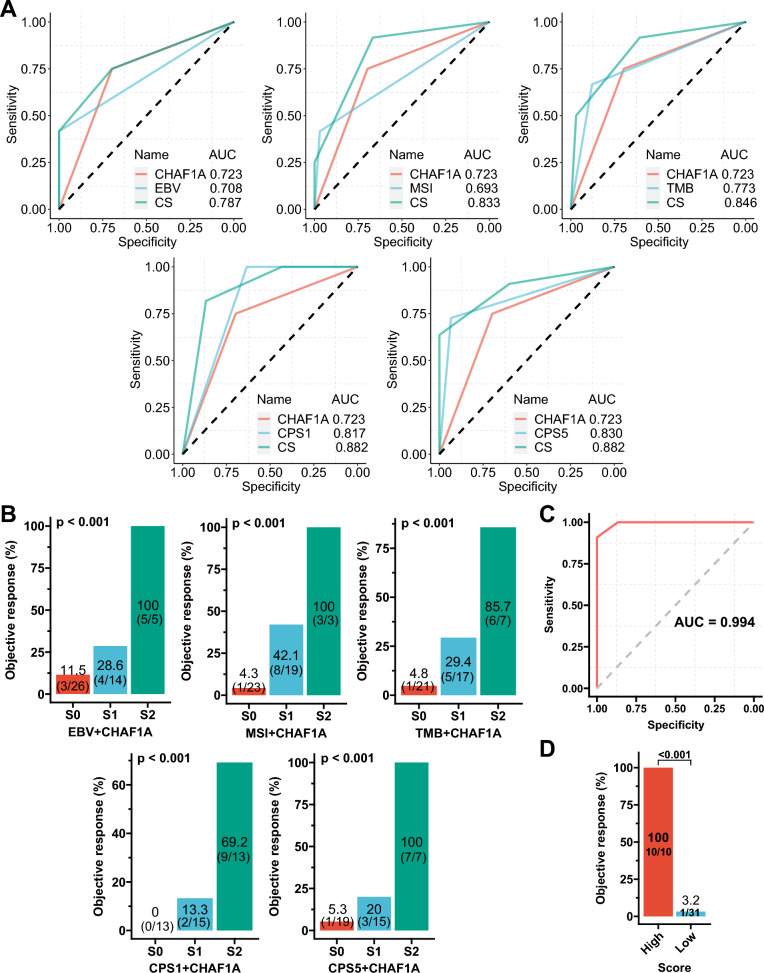
Immunotherapy response and the combined scoring of CHAF1A with classic biomarkers in gastric cancer. **A**: The ROC curves for response prediction by CHAF1A expression, classic biomarkers, and their pairwise combinations. **B:** Objective response rate (ORR) according to the combined scoring of CHAF1A with classic biomarkers. **C**: The ROC curve for response prediction by the combined scoring of all biomarkers. **D**: ORR according to the combined scoring of all biomarkers. *ROC* receiver operating characteristic curve, *AUC* the areas under the ROC curves, *CS* combined scoring, *MSI* microsatellite instability, *TMB* tumor mutation burden, *CPS1 or CPS5* combined positive score of PD-L1 with a cutoff value of 1 or 5; S0, S1 and S2: score 0, 1 and 2

Immunotherapy responses are available for the NCT#02589496 cohort. The AUC of the ROC curves for predicting the immunotherapy response were 0.723, 0.708, 0.693, 0.773, 0.817, and 0.830 for mRNA expression of *CHAF1A*, EBV, MSI, TMB, CPS1, and CPS5, respectively. The AUC values were 0.787, 0.833, 0.846, 0.882, and 0.882 for the CS of CHAF1A expression with EBV, MSI, TMB, CPS1, and CPS5, respectively (Fig. 7A). The ORR of patients with a score of 0, 1, and 2 were 11.5, 28.6, and 100% (*p* < 0.001), 4.3, 42.1, and 100% (*p* < 0.001), 4.8, 29.4, and 85.7% (*p* < 0.001), 0, 13.3, and 69.2% (*p* < 0.001), and 5.3, 20, and 100% (*p* < 0.001) for the CS of CHAF1A expression with EBV, MSI, TMB, CPS1, and CPS5, respectively (Fig. 7B). A model that included all of these biomarkers was constructed with an AUC of 0.994 to predict the response (Fig. 7C). The optimal score threshold of this model for the maximum ROC curve value was used to divide the high and low scores. The ORR of patients with high and low scores were 100 and 3.2%, respectively (*p* < 0.001; Fig. 7D).

### Validation of the role of CHAF1A in a different immunotherapy cohort

Because other GC cohorts for immunotherapy are not available, the IMvigor210 cohort of mUC, which has been widely used to investigate biomarkers of immunotherapy [24], was used to validate the role of CHAF1A. In this cohort, TMB, TNB, and the immune phenotype (IP), were available, and PD-L1 expression on tumor cells (TC) and immune cells (IC) was stained and evaluated as IC0/TC0 (< 1%), IC1/TC1 (≥ 1% and < 5%), or IC2/TC2 (≥ 5%). We converted IC0/TC0 to 0 (PD-L1 negative) and IC1/TC1 and IC2/TC2 to 1 (PD-L1 positive). The CS of CHAF1A expression with these biomarkers were determined. The ORR of patients with a score of 0, 1, and 2 were 18.3, 32.9, and 53.3% (*p* = 0.002), 14, 31.3, and 64% (*p* < 0.001), 12, 41.8, and 68.2% (*p* < 0.001), 10.1, 22, and 51.4 (*p* < 0.001), and 17.7, 29.5, and 63.6% (*p* < 0.001) for the CS of CHAF1A expression with IP, TMB, TNB, IC, and TC, respectively (Additional file 1: Table. S2).

OS data were also available for this cohort. The median OS of patients with a score of 0, 1, and 2 were 8.7 months, 15.9 months, and NR for the combination of CHAF1A with IP (p = 2e-04); 9.0 months, 13.4 months, and NR for the combination of CHAF1A with TMB (p = 0.0002); 8.2 months, 21.2 months, and NR (no events in 6 patients) for the combination of CHAF1A with TNB (p < 0.0001); 7.9 months, 10.5 months, and NR for the combination of CHAF1A with IC (p < 0.0001); and 9.3 months, 13.4 months, and NR for the combination of CHAF1A with TC (p = 0.028), respectively (Additional file 2: Fig. S1).

## Discussion

Although regimens containing ICIs has become the first-line treatment of GC, there is still no evidence to suggest that patients with GC can benefit from second-line immunotherapy, and the ORR of third-line immunotherapy for GC is extremely low. Recently, owing to unconfirmed clinical benefits, pembrolizumab has been withdrawn as a third-line treatment for GC [25]. Some biomarkers such as EBV infection, MSI, TMB, and PD-L1 expression have been found to predict immunotherapy efficacy, while studies have reported inconsistent results [4]. There is still a need to further improve the prediction of immunotherapy response, especially in the second/third-line treatment of GC.

In this study, we identified an EBV-associated gene, *CHAF1A*, which is upregulated by EBV infection; both its mRNA and protein expression predicted EBV infection in GC. Moreover, CHAF1A alone could predict the prognosis of patients with GC well, but its combination with classic biomarkers, including MSI and TMB, further improved prognostic stratification. Importantly, CHAF1A was a response predictor of immunotherapy for GC, and CS of CHAF1A with EBV, MSI, TMB, or PD-L1 expression further stratified the ORR, which increased with an increase in CS. When all these biomarkers were available, a corresponding model could perfectly predict the response, with an AUC of 0.994. These results indicated that CHAF1A may be a novel immunotherapy biomarker.

CHAF1A is a subunit of chromatin assembly factor-1 (CAF-1), an H3-H4 histone chaperone [26]. In addition to its epigenetic role, functional versatility of CHAF1A has been reported in GC. The CHAF1A/TCF4 complex directly binds to the promoter regions of c-MYC and CCND1 to enhance their transcriptional activation, thereby promoting gastric carcinogenesis [27]. Interestingly, HP infection in GC upregulates CHAF1A expression, which is dependent on the binding of specific protein 1 to the CHAF1A promoter [27]. Recently, CHAF1A is reported to play a role in the infection of human immunodeficiency virus 1 (HIV-1) and be critical in the establishment and maintenance of HIV-1 latency [28, 29]. In our study, we revealed that EBV infection induced CHAF1A expression, and GSEA suggested that CHAF1A was associated with many infection signaling pathways involving both bacteria and viruses. In particular, the genes involved in the viral carcinogenesis pathway were significantly enriched in the high CHAF1A expression group. Together, these results indicated that CHAF1A participates in pathogen infection and mediates the oncogenic roles of some pathogens.

The role of CHAF1A in anti-cancer immunity remains unclear. Recently, regulators similar to CHAF1A in chromatin organization and remodeling have been reported to play critical roles in anticancer immunity, and have therefore become promising targets for cancer treatment [30, 31]. Our GSEA showed that CHAF1A was associated with many DNA repair and metabolic pathways. Defective DNA repair increases genomic mutations and instability, which may promote the production of tumor neoantigens and subsequently increase the immunogenicity of tumor cells [32]. It is also well known that abnormal metabolism in cells of the tumor microenvironment driven by metabolic reprogramming is closely linked to anticancer immunity [33]. These findings, together with our results showing positive correlations between CHAF1A and MSI, TMB, and immune cell infiltration, suggest that CHAF1A activates anticancer immunity.

Recently, chromatin regulators are revealed to significantly impact tumor response to immunotherapy. The SWItch/sucrose non-fermentable (SWI/SNF) chromatin remodeling complex plays a central role in the coordination of T cell activation and exhaustion [34]. Inhibition of SWI/SNF results in improved antitumor control, both alone and in combination with immunotherapy [35]. Genomic alterations in SWI/SNF also affect the response to immunotherapy, and are therefore promising predictive biomarkers [36]. In our study, the expression of CHAF1A showed the potential to predict immunotherapy response. Similar biomarkers have been widely reported in recent years. However, few of these have been verified in prospective studies, and inconsistent results are concerning. Classic biomarkers such as MSI, TMB, and PD-L1 remain the main basis for clinical decisions. Importantly, CHAF1A was found to be a favorable assistant for the classic biomarkers. The CS of CHAF1A expression with classic biomarkers improved the stratification of both prognosis and immunotherapy outcomes, indicating the possibility of optimizing the use of current biomarkers.

Our study has several limitations. First, the mechanisms by which EBV upregulates CHAF1A expression and the subsequent biological effects of CHAF1A overexpression after EBV infection remain unknown. Second, the mechanisms by which CHAF1A regulates anticancer immunity and determines immunotherapy outcomes remain unclear. Moreover, only one GC cohort undergoing immunotherapy was available for this study, and more such cohorts are required to validate our findings. Finally, prospective validations of a biomarker is necessary.

In conclusion, CHAF1A, a novel biomarker associated with EBV infection, was revealed to be a predictor for prognosis and immunotherapy response in GC. Particularly, CHAF1A had been shown to optimize clinical practice based on current biomarkers by improving their effects. Further validation and research on detail mechanisms are required.

### Supplementary Information

**Additional file 1: Table S1.** Clinicopathological characteristics. **Table S2.** Response to immunotherapy according to the combination of CHAF1A with classic biomarkers in the IMvigor210 cohort.

**Additional file 2: Figure S1.** Overall survival stratified by the combined scoring of CHAF1A with classic biomarkers in the IMvigor210 cohort. A-E: Overall survival by the combined scoring of CHAF1A expression with tumor mutation burden (A), tumor neoantigen burde (B), immune phenotype (C), PD-L1 expression on immune cells (D) and PD-L1 expression on tumor cells (E).

## Data Availability

All data relevant to the study that are not in the article and supplementary material are available from the corresponding author on reasonable request.

## Abbreviations

GC: Gastric cancer
PD-1: Programmed cell death receptor-1
ICIs: Immune checkpoint inhibitors
OS: Overall survival
PFS: Progression-free survival
CPS: Combined positive score
TCGA: The Cancer Genome Atlas
CIN: Chromosomal instability
EBV: Epstein-Barr virus
GS: Genomically stable
MSI: Microsatellite instability
EBER-ISH: EBV-encoded small RNA in situ hybridization
ORR: Overall response rate
AHJU: Affiliated Hospital of Jiangsu University
IHC: Immunohistochemistry
ACRG: Asian Cancer Research Group
mUC: Urothelial cancer
TMB: Tumor mutation burden
TNB: Tumor neoantigen burden
MSS: Microsatellite stability
DEGs: Differentially expressed genes
HRs: Hazard ratios
CIs: Confidence intervals
GO: Gene Ontology
WB: Western blot
mIF: Multiple-immunofluorescence
CS: Combined score
GSEA: Gene set enrichment analysis
HP: Helicobacter pylori
CAF-1: Assembly factor-1
HIV-1: Human immunodeficiency virus 1
