# Supplementary material for: A novel biomarker associated with EBV infection improves response prediction of immunotherapy in gastric cancer
Source: J Transl Med. 2024 Jan 22;22:90. doi: 10.1186/s12967-024-04859-8 (PMC10804498; doi:10.1186/s12967-024-04859-8)
Supplement: Supplementary file 1 — Additional file 1: Table S1. Clinicopathological characteristics. Table S2. Response to immunotherapy according to the combination of CHAF1A with classic biomarkers in the IMvigor210 cohort. [file 12967_2024_4859_MOESM1_ESM.docx]

**Table S1.** Clinicopathological characteristics.

| **Variable** | | **EBV** | | **P value** |
| --- | --- | --- | --- | --- |
|  |  | **Negative** | **Positive** |  |
| **AHJU cohort** | | | | |
| Sex | Women | 6 (28.6) | 0 (0.0) | 0.173 |
|  | Men | 15 (71.4) | 5 (100.0) |  |
| Age | <65 years | 4 (19.0) | 3 (60.0) | 0.064 |
|  | ≥65 years | 17 (81.0) | 2 (40.0) |  |
| Histologic grade | Ⅰ/Ⅱ | 3 (14.3) | 3 (60.0) | 0.029 |
|  | Ⅲ/Ⅳ | 18 (85.7) | 2 (40.0) |  |
| Stage | Ⅰ/Ⅱ | 18 (85.7) | 3 (60.0) | 0.190 |
|  | Ⅲ/Ⅳ | 3 (14.3) | 2 (40.0) |  |
| **ACRG cohort** | | | | |
| Sex | Women | 88 (34.2) | 2 (11.1) | 0.043 |
|  | Men | 169 (65.8) | 16 (88.9) |  |
| Age | <65 years | 139 (54.1) | 10 (55.6) | 0.904 |
|  | ≥65 years | 118 (45.9) | 8 (44.4) |  |
| Histologic grade | Ⅰ/Ⅱ | 113 (44.0) | 3 (16.7) | 0.023 |
|  | Ⅲ/Ⅳ | 144 (56.0) | 15 (83.3) |  |
| Stage | Ⅰ/Ⅱ | 106 (41.2) | 6 (33.3) | 0.509 |
|  | Ⅲ/Ⅳ | 151 (58.8) | 12 (66.7) |  |
| **TCGA cohort** | | | | |
| Sex | Women | 115 (35.7) | 4 (14.8) | 0.028 |
|  | Men | 207 (64.3) | 23 (85.2) |  |
| Age | <65 years | 131 (40.9) | 14 (51.9) | 0.269 |
|  | ≥65 years | 189 (59.1) | 13 (48.1) |  |
| Histologic grade | Ⅰ/Ⅱ | 135 (43.1) | 2 (7.4) | <0.001 |
|  | Ⅲ/Ⅳ | 178 (56.9) | 25 (92.6) |  |
| Stage | Ⅰ/Ⅱ | 147 (47.6) | 8 (29.6) | 0.073 |
|  | Ⅲ/Ⅳ | 162 (52.4) | 19 (70.4) |  |
| **NCT#02589496 cohort** | | | | |
| Sex | Women | 14 (28.0) | 2 (40.0) | 0.573 |
|  | Men | 36 (72.0) | 3 (60.0) |  |
| Histologic grade | Ⅰ/Ⅱ | 18 (40.9) | 2 (33.3) | 0.722 |
|  | Ⅲ/Ⅳ | 26 (59.1) | 4 (66.7) |  |

AHJU: Affiliated Hospital of Jiangsu University; ACRG: Asian Cancer Research Group; TCGA: The Cancer Genome Atlas.

**Table S2.** Response to immunotherapy according to the combination of CHAF1A with classic biomarkers in the IMvigor210 cohort.

| **Biomarker** | **Response** | **Combined score** | | | **P value** |
| --- | --- | --- | --- | --- | --- |
|  |  | **2** | **1** | **0** |  |
| IP+CHAF1A | No | 7 (46.7) | 51 (67.1) | 125 (81.7) | 0.002 |
|  | Yes | 8 (53.3) | 25 (32.9) | 28 (18.3) |  |
| TMB+CHAF1A | No | 9 (36.0) | 55 (68.8) | 166 (86.0) | <0.001 |
|  | Yes | 16 (64.0) | 25 (31.3) | 27 (14.0) |  |
| TNB+CHAF1A | No | 7 (31.8) | 39 (58.2) | 184 (88.0) | <0.001 |
|  | Yes | 15 (68.2) | 28 (41.8) | 25 (12.0) |  |
| IC+CHAF1A | No | 18 (48.6) | 149 (78.0) | 62 (89.9) | <0.001 |
|  | Yes | 19 (51.4) | 42 (22.0) | 7 (10.1) |  |
| TC+CHAF1A | No | 4 (36.4) | 62 (70.5) | 163 (82.3) | <0.001 |
|  | Yes | 7 (63.6) | 26 (29.5) | 35 (17.7) |  |

IP: Immune phenotype; TMB: tumor mutation burden; TNB: tumor neoantigen burden; IC: PD-L1 expression on immune cells; TC: PD-L1 expression on tumor cells; S0, S1 and S2: score 0, 1 and 2.
