# Supplementary figures and images for: A novel biomarker associated with EBV infection improves response prediction of immunotherapy in gastric cancer
Source: J Transl Med. 2024 Jan 22;22:90. doi: 10.1186/s12967-024-04859-8 (PMC10804498; doi:10.1186/s12967-024-04859-8)

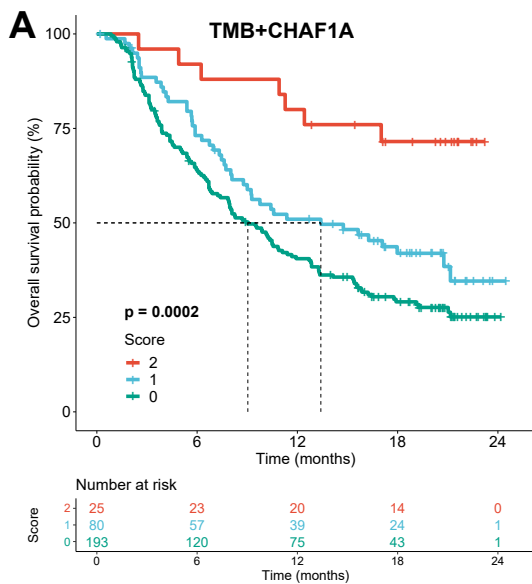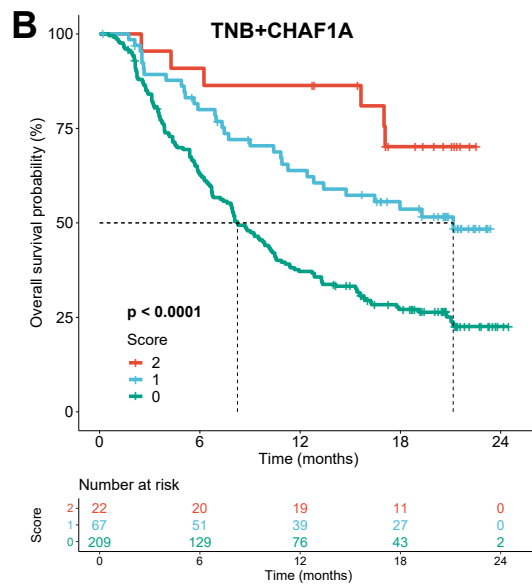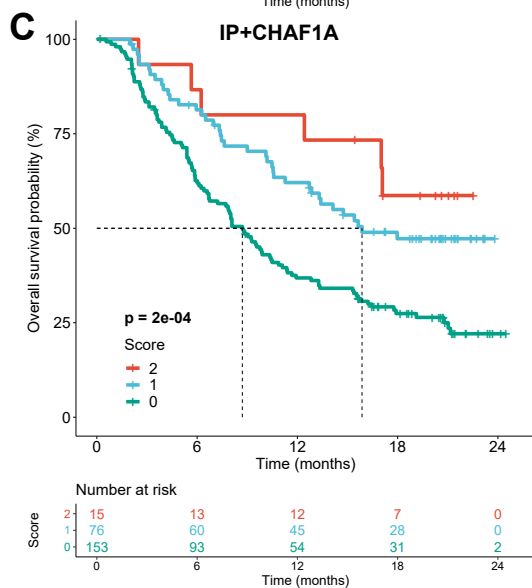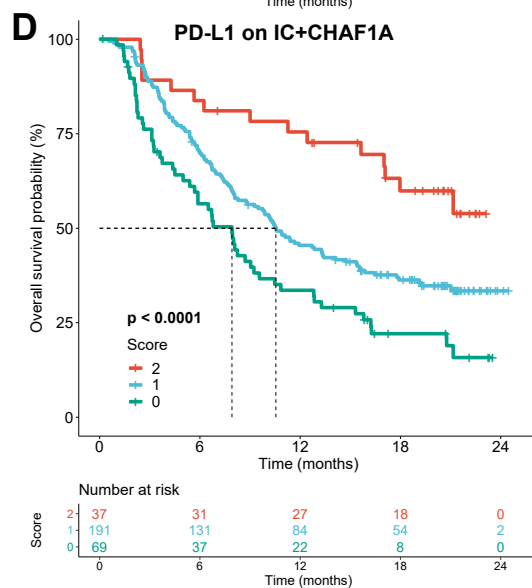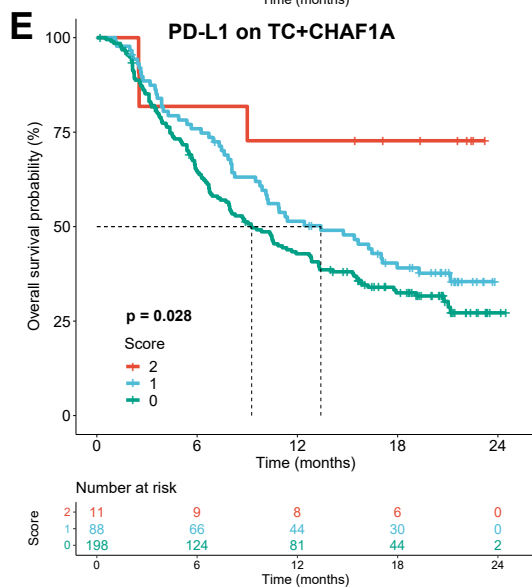

Supplement: Supplementary file 2 — Additional file 2: Figure S1. Overall survival stratified by the combined scoring of CHAF1A with classic biomarkers in the IMvigor210 cohort. A-E: Overall survival by the combined scoring of CHAF1A expression with tumor mutation burden (A), tumor neoantigen burde (B), immune phenotype (C), PD-L1 expression on immune cells (D) and PD-L1 expression on tumor cells (E). [file 12967_2024_4859_MOESM2_ESM.pdf]
